# Dysfunctional loop ileostomy after low anterior resection for rectal cancer in the presence of Meckel’s diverticulum: a case report

**DOI:** 10.1186/s13256-015-0673-x

**Published:** 2015-09-10

**Authors:** Konstadinos G. Spiridakis, Eleftherios E. Sfakianakis, Manthos E. Flamourakis, Margetousakis C. Theodoros, Efstathios K. Rahmanis, Evaggelia M. Polychronaki, George E. Kostakis, Theodoros G. Papadakis, Manousos S. Hristodoulakis

**Affiliations:** Department of Surgery, General Hospital of Heraklion Crete, Venizelio, Greece

## Abstract

**Introduction:**

A temporary protective loop ileostomy is a routine procedure to protect the anastomosis in patients who undergo low anterior resection for rectal cancer. The aim of this case report is to present a rare complication caused by Meckel’s diverticulum.

**Case presentation:**

We describe a case of a 71-year-old white man with dysfunctional ileostomy after low anterior resection for rectal cancer due to adhesions and pressing effects of Meckel’s diverticulum near the ileostomy site, which led to volvulus of his small intestine and obstruction. As a result he underwent a supplementary operation to resolve this complication by Meckel’s diverticulum resection.

**Conclusions:**

During a low anterior resection for rectal cancer and a protective ileostomy procedure the presence of Meckel’s diverticulum should not be ignored. Our proposal is the primary resection of Meckel’s diverticulum as the best surgical choice; according to the limited international literature on such cases we report a possible alternative to a protective ileostomy by creating a conduit using Meckel’s diverticulum as a stoma.

## Introduction

A temporary protective ileostomy is a routine procedure for patients who undergo a low anterior resection for rectal cancer with anastomosis less than 5cm from the anal verge. We present a rare case of dysfunctional temporary loop ileostomy due to adhesions and volvulus caused by Meckel’s diverticulum (MD) which was primarily ignored.

## Case presentation

A 71-year-old white man was diagnosed with rectal adenocarcinoma 5cm from the anal verge. Computed tomography (CT) and magnetic resonance imaging (MRI) highlighted circumferential thickened rectal mucosa. The tumor was considered to be resectable and he underwent a low anterior resection with an end-to-end anastomosis 2cm from the rectal verge in which circular staplers EEA™ 28mm were used. A protective ileostomy was created. A Meckel’s diverticulum was encountered near the site of the ileostomy, 7cm away from the afferent limb of the protective ileostomy, without any further action.

During the early postoperative period his progress was satisfactory and the loop ileostomy was functional, but on postoperative day (POD) 4 the ileostomy appeared dysfunctional and he presented symptoms of incomplete ileus.

During POD 5 a Foley catheter was inserted in the ileostomy and remained there for 2 days. The ileostomy started to be more functional, but after the catheter was removed, obstruction symptoms reappeared. An endoscopy in the ileostomy revealed mechanical obstruction of the ileostomy by adjacent bowel loop and possible volvulus of the ileostomy loop.

**Fig. 1:**
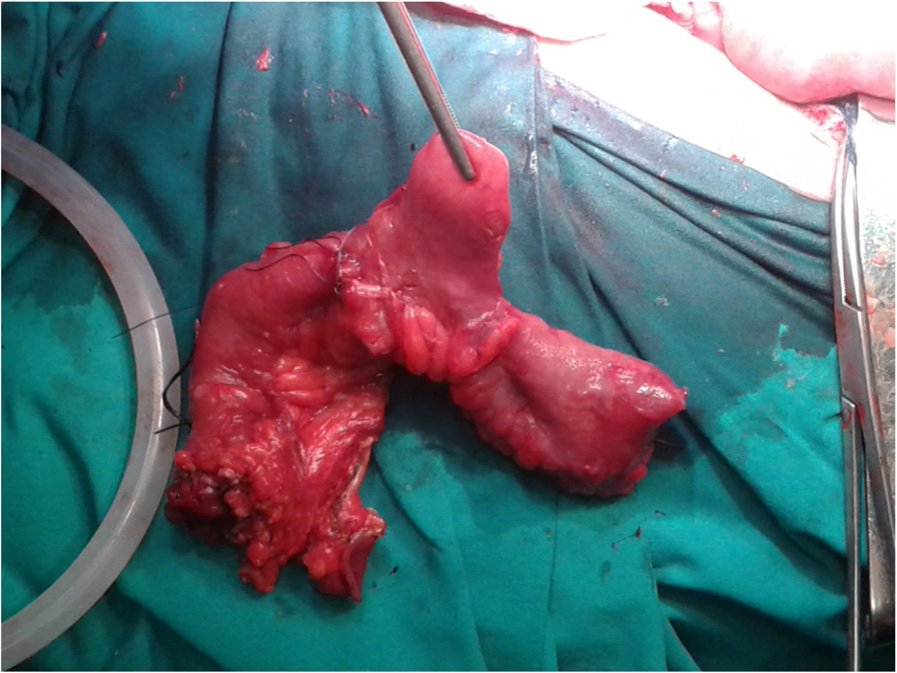
Resected specimen of Meckel’s diverticulum with part of small bowel

In consequence, the patient underwent a supplementary operation, in which we found volvulus of loop ileostomy as a result of adhesions and pressing effects of MD, which was encountered close to the ileostomy. Adhesiolysis and a resection of the MD (Fig. 1) were performed as well as closure of the ileostomy. His postoperative progress was optimal and he was discharged on POD 8 after the closure of the ileostomy.

## Discussion

Colorectal cancer represents 9% of all cancers around the globe. It is the third most common cause of death in the USA [1]. In 2008, 148,000 new cases of colorectal cancer were recorded [2]. There are similar incidence rates for cancer of the colon in both sexes, and a slight male predominance for rectal cancer. In the UK, colorectal cancer is the second most common cause of death, with 20,000 new cases every year [3]. In addition, in many countries around the world, including European countries, rates of colorectal cancer are high. Diagnosis and staging of rectal cancer includes CT scan, MRI (in some cases), endoscopy and biopsies.

MD was first described in 1803 as a vestigial remnant of the omphalomesenteric duct. It is often encountered incidentally during appendectomy and it is associated with serious complications such as obstruction, intussusception, perforation, diverticulitis and gastrointestinal bleeding [4].

In low anterior resection for rectal cancer, protective ileostomy is strongly suggested when anastomosis is performed less than 5cm from the anal verge [5]. The construction of a protective stoma significantly reduces the incidence of anastomotic leakage and re-operation [5, 6].

Our case report is about a rare case of dysfunctional ileostomy in which the MD caused volvulus of the small intestine near the ileostomy and this in combination with its adhesions led to bowel obstruction and, as a result, dysfunction of the protective ileostomy.

Research of the limited international literature on cases similar to this case report revealed a report of the use of MD as a urinary conduit [7]. We found a case report by Sheena *et al*. that used MD as an alternative conduit to a defunctioning ileostomy, which reported satisfactory results [3].

## Conclusions

In conclusion, during a low anterior resection for rectal cancer and a protective ileostomy procedure, the presence of MD should never be ignored. Our proposal is the primary resection of MD as the best surgical choice for avoiding the complication we have described in our case report.

## Consent

Written informed consent was obtained from the patient for publication of this case report and any accompanying images. A copy of the written consent is available for review by the Editor-in-Chief of this journal.

## Abbreviations

CT: Computed tomography
MD: Meckel’s diverticulum
MRI: Magnetic resonance imaging
POD: Postoperative day
